# Specific leaf area of European Larch (*Larix decidua* Mill.)

**DOI:** 10.1007/s00468-016-1361-1

**Published:** 2016-02-16

**Authors:** Helga Fellner, Gerald F. Dirnberger, Hubert Sterba

**Affiliations:** Department of Forest and Soil Science, University of Natural Resources and Life Sciences (BOKU), Peter Jordan Strasse 82, 1190 Vienna, Austria

**Keywords:** *Larix decidua* Mill., Specific leaf area (SLA), Mixed stands, European larch, Branch height, Canopy depth

## Abstract

*****Key message***:**

**The specific leaf area of European larch depends on branch height and canopy depth, indicating that both, the effect of hydraulic limitations and low water potentials in greater branch heights, and light availability affect specific leaf area.**

**Abstract:**

Specific leaf area (SLA) is defined as the ratio between projected leaf area and needle dry mass. It often serves as parameter in ecosystem modelling as well as indicator for potential growth rate. We explore the SLA of European larch (*Larix decidua*) and the most important factors which have an influence on it. Data were collected from eight stands in Styria, Austria. The stands varied in age, elevation and species mixture. Four stands were pure larch stands with only minor proportions of Norway spruce (*Picea abies*), whereas the other four were mixed stands of larch and spruce. In each stand 15 representative sample trees were felled. The crown of each sample tree was divided into three sections of equal length and in each section a random sample of needles was taken for determining projected leaf area and dry mass of 50 needles. The mean SLA of larch was established to be 117 cm^2^ g^−1^ with a standard deviation of ±27.9 cm^2^ g^−1^. SLA varies within the crown, but neither between different mixtures nor years of observation nor social position of the trees. A mixed-effects model, with the plots as random effect, revealed that SLA of larch decreased with increasing branch height (*p* = 0.0012) and increased with increasing canopy depth (*p* = 0.029). We conclude that both the hydraulic limitations due to low water potentials in greater branch heights and light availability affect specific leaf area.

## Introduction

Due to its characteristics and ecological value, larch is a highly prevailing tree species especially in mountainous areas. European larch (*Larix decidua* Mill.) can be found in the northern boreal forests from the lowlands to the mountains, whereas in the southern extension it occurs only in the mountainous regions. It is a significant timberline species in Central Europe. In Austria it accounts for 4 % of tree species distribution, in elevations above 900 m; however, its proportion is already 8 %. This still may sound little compared to the 53 % domination of Norway spruce, but the presence of European larch is an important admixture for enrichment of the species diversity and stabilisation of mountain forests with regard to landslides. It serves as a resilient tree species in protective forests as well as pioneer tree species for re-colonisation on large natural disturbances. Compared to other conifers, larch is very shade intolerant, but can cope with poor soils and little water availability (Schober 1949; Mayer 1977; Englisch et al. 2011; Bundesforschungszentrum für Wald (BFW) 2013).

Another feature of European larch is that it has long shoot needles as well as short shoot needles. They look different in their phenology. Needles on short shoots are arranged in little clusters of 20–40 needles, whereas needles on long shoots are arranged alternately. Needles are shed in autumn, which is unique among European conifers (Geburek 2002).

Specific leaf area (SLA) is defined as the ratio between projected leaf area and needle dry mass (cm^2^ g^−1^). Research shows that SLA reacts very sensitively to changes of the availability of resources, e.g. light, humidity and nutrition (Gholz et al. 1976; Smith et al. 1981; Matyssek 1986; Klinka et al. 1992; Wilson et al. 1999; Marshall and Monserud 2003; White and Scott 2006; Poorter et al. 2009).

SLA is important for estimating the leaf area of whole trees (Monserud and Marshall 1999; Xiao et al. 2006) and it is positively and linearly correlated to relative growth rate (Poorter et al. 2009). Although it is an often used parameter for ecosystem functions, its reciprocal value, leaf mass per area (LMA), is also used because it correlates positively and linearly with additional investments in the leaf thickness (Poorter et al. 2009). Specific leaf weight is also highly correlated with the annual photosynthetic rate (Oren et al. 1986; Wilson et al. 1999; Cornelissen et al. 2003; Poorter et al. 2009).

SLA increases with decreasing light conditions, and thus with canopy depth. Stand structure has a similar influence on the light conditions, and therefore also affects SLA (Del Rio and Berg 1979; Abrams and Kubiske 1990; Chen and Klinka 1998; Tardieu et al. 1999; Bond et al. 1999; Nagel and O’Hara 2001; Poorter et al. 2006).

Less shade tolerant species have a lower SLA and are less flexible in physical leaf properties than shade tolerant tree species (Abrams and Kubiske 1990; Smith et al. 1991; Chen et al. 1996; Chen 1997; Bond et al. 1999; Marshall and Monserud 2003; Xiao et al. 2006). Conversely, high SLA can be found at shade tolerant tree species (Cornelissen et al. 2003; Lichtenthaler et al. 2007; Perrin and Mitchell 2013).

Light conditions are not the only reason for high or low SLA; it also depends on the longevity of leaves (Gholz et al. 1976; Del Rio and Berg 1979) and differs between evergreen and deciduous trees (Gower and Richards 1990; Withington et al. 2006).

The availability of nutrients also influences SLA. In environments that are well-supplied with nutrients, species tend to have a higher SLA than in nutrient-poor environments (Pierce et al. 1994; Cornelissen et al. 2003; White and Scott 2006; Poorter et al. 2009). Withington et al. (2006) found positive relationships between SLA, leaf nitrogen and tissue density of the roots while there was a negative relationship with root production when studying six *Pinacea*, among them one plot with European larch.

SLA has also been found to be affected by hydraulic limitations and lower water potentials in greater branch heights, due to the pull of the water column (Chen 1997; Tardieu et al. 1999; Nagel and O’Hara 2001; Marshall and Monserud 2003; Koch et al. 2004).

Compared to other conifer tree species such as Norway spruce (*Picea abies* (L.) H. Karst.) with a mean SLA of 45.8 cm^2^ g^−1^ (Oleksyn et al. 1998) to 50 cm^2^ g^−1^ (Hager and Sterba 1985), Scots pine (*Pinus sylvestris* L.) with 43.8 cm^2^ g^−1^ (Xiao et al. 2006), Douglas-fir (*Pseudotsuga menziesii* (Mirb.) Franco var. glauca) with 34.3 cm^2^ g^−1^, Western white pine (*Pinus monticola* Dougl. Ex D. Don) with 41.4 cm^2^ g^−1^ and Ponderosa pine (*Pinus ponderosa* Dougl. Ex. P. & C. Laws.) with 25.8 cm^2^ g^−1^ (Marshall and Monserud 2003), the specific leaf area of European larch is expected to be two to four times higher (see e.g. Gower and Richards 1990; Wieser et al. 2013). Conversely for two more flat-needles species Lichtenthaler et al. (2007) report SLAs of 60–80 cm^2^ g^−1^ for young *Abies alba* (Mill.) and Perrin and Mitchell (2013) even 100–200 cm^2^ g^−1^ for *Taxus baccata* (L.) saplings.

The objective of this investigation is (1) to find the average SLA of larch, and (2) to investigate the dependence of SLA on site-, stand- and tree characteristics and especially try to evaluate its dependence on light conditions versus its dependence on hydrologic limitations.

## Materials and methods

### Study area and study design

The observed plots are located in the northern part of the eastern intermediate Alps near Leoben in Styria, Austria. The coordinates are 47°26′ east latitude and 15°05′ north longitude at an altitude of 900–1300 m above sea level. The mean annual temperature is 6.1 °C and the mean annual precipitation is 1000 mm (ZAMG 2014—observation period between 1971 and 2000). The maximum rainfall occurs in July. The soils are mostly poor podzolic brown soils (Kilian et al. 1994). All the stands were located in steep terrain (slope 50–70 %) and exposed to Northwest to West. The mean annual volume increment at age 100 [estimated according to Marschall (1975)], only varies between 8 and 9 m^3^ per year and hectare, indicating medium site quality.

Data were collected in four stands during the growing season of 2012 and in four more stands in 2013. The stands varied in age. In each year, 2012 and 2013, we selected two nearly pure larch stands, and two other stands with mixtures of Norway spruce and European larch. In each stand a plot was established (for plot size see Table 1). In these plots a full inventory was performed, determining tree species, diameter at breast height (DBH), tree height and height to the crown base.

The larches of each plot were classified into three social classes (SOC in Abbreviations) of equal frequency (dominated, intermediate and dominant) by their DBH. Five representative trees per class were selected, excluding trees on plot edges or with irregular crown shape. Overall, this resulted in 15 sample trees per plot. In sum, there were 120 sample trees for both years (60 in mixed stands and 60 in pure stands). The selection process was crucial to ensure a broad variation of growing conditions of the collected needles for further analyses. These sample trees were felled and age was determined by counting the tree rings of the stump. The crown of each sample tree was divided into three sections of equal length (CS in Abbreviations). From each crown third a representative branch was chosen and a handful twigs were collected. From these twigs 50 needles were picked for weighing and determining the leaf area. In 2013 we also collected short and long shoot needles separately from the same trees and in the same locations to test if there were differences between the two kinds of needles in regard of SLA. All 50 needles together were scanned immediately after their collection in the field and surveyed with a raster graphics editor [Adobe (2014) Photoshop CS4^®^] subsequently in the laboratory, resulting in the projected leaf area. These needle samples were dried to constant mass at 105 °C and weighed (Table 1).

**Table 1:** Stand level characteristics and plot attributes

Plot number	Year	Stand-type	Plot size (ha)	Age (years)	QMD (cm)	*H*_dom_ (m)	Stocking degree	Norway spruce (%)
2	2012	Mixed	0.266	44 (±5)	27.3	21.5	1.162	53.6
3	2012	Pure	0.250	46 (±5)	22.8	21.2	1.731	13.2
5	2013	Mixed	1.166	98 (±16)	36.4	35.0	0.768	33.5
6	2013	Pure	0.681	92 (±3)	30.7	31.7	0.891	12.0
8	2012	Mixed	0.328	131 (±13)	44.5	33.5	1.263	49.3
9	2012	Pure	1.154	147 (±6)	46.0	39.3	0.874	14.2
11	2013	Mixed	1.289	186 (±6)	48.3	31.8	0.994	33.1
12	2013	Pure	1.110	96 (±9)	35.6	32.2	0.790	0.2

Note that the missing plot numbers, referring to the established pure Norway spruce stands, were not used in this investigation. The age is the average age of the sample trees, in brackets, the standard deviation. QMD is the quadratic mean diameter. *H*_dom_ the dominant height, i.e. the mean height of the largest 100 trees per ha. Stocking degree is calculated according to the yield tables by Marschall (1975). The proportion of spruce is in % of the crown projection area in the layer above 60 % of the maximum tree height

**Fig. 1:**
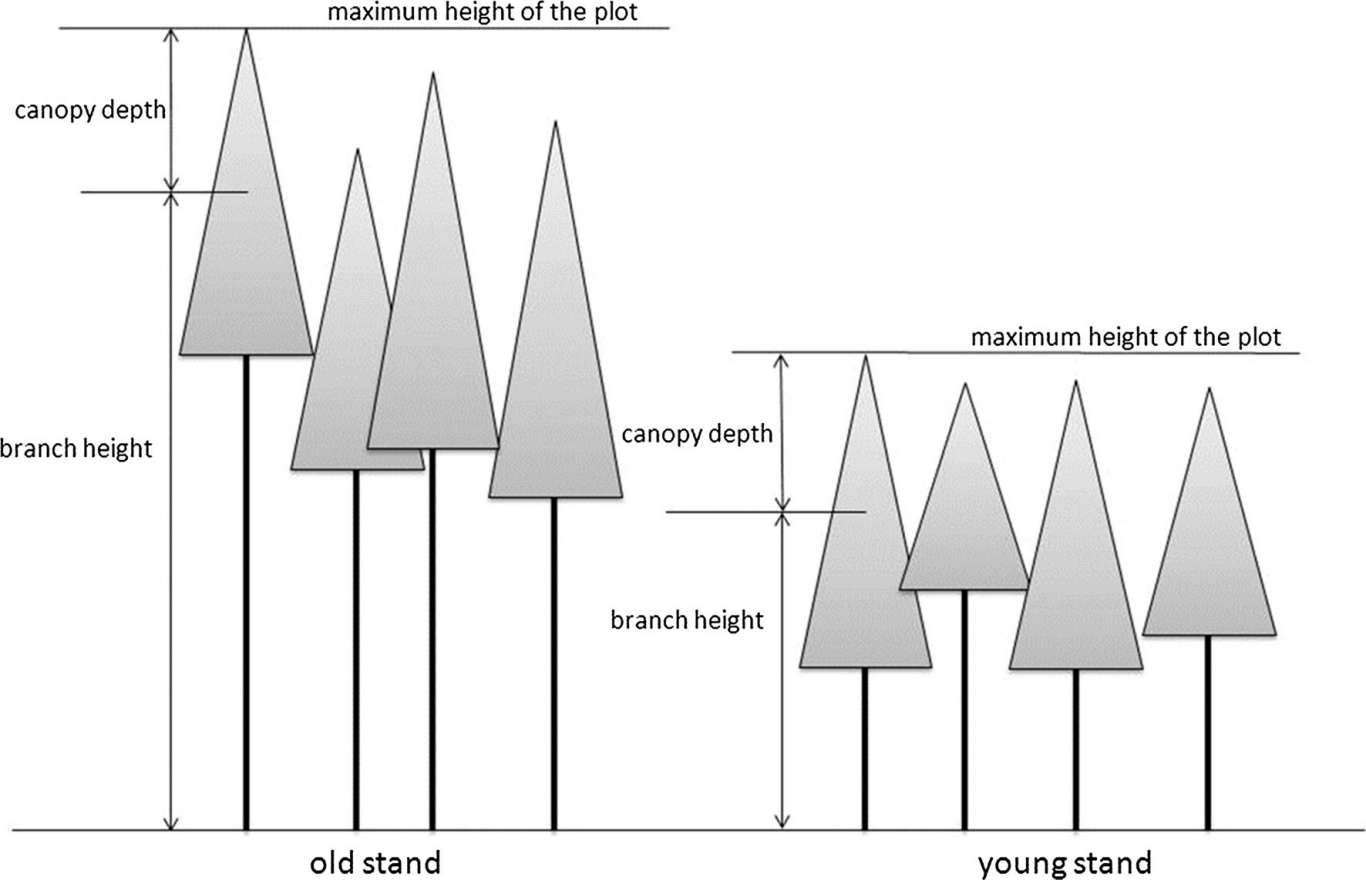
Definition of branch height and canopy depth

For describing the light availability of each sample we used the following calculation. As shown in Fig. 1, first, the height of the largest tree in each plot was searched. From this maximum tree height, the branch height of the sampled branch in each individual sample tree was subtracted, resulting in the canopy depth as a proxy for the light availability.

### Statistical analysis

After data collection and calculation of the SLA for all samples, a series of tests were conducted. First of all, a pairwise *t* test was run to search for differences of the SLA of long and short shoot needles. Then one-way ANOVAs were performed for detecting SLA differences between the main factors, year of sampling, stand type (pure or mixed), social position of the tree, and crown section within the trees. Because of the unbalanced and hierarchical data structure we used mixed-effects models approach for testing for influential variables all together, with the plots and the trees within the plot as random effects (*u*) and the fixed effects (*x*) altitude above sea level, year of sampling, quadratic mean diameter, age and mixture, DBH, tree height, branch height and canopy depth.

1 $$y = a_{0} + a_{1} x_{1} + a_{2} x_{2} + \cdots + a_{n} x_{n} + b_{1} x_{1} x_{2} + b_{2} x_{1} x_{3} + \cdots + b_{nn} x_{n - 1} x_{n} + u_{\text{tree}} + u_{\text{plot}}$$

where *a*_*i*_ are the coefficients for the main effects, *x*_*i*_, and *b*_*i*_ the coefficients for the respective interaction terms, *x*_*i*_*x*_*j*_.

All statistical calculations were performed in the program R (R Development Core Team 2013), the mixed-effects models were calculated with the function *lme*. For comparing the different mixed-effects models we used the procedure after Pinheiro and Bates (2004, p. 159), where a likelihood ratio test was used.

## Results

The overall arithmetic mean SLA resulted in 117 cm^2^ g^−1^ with a standard deviation of ±27.9 cm^2^ g^−1^ (Table 2).

**Table 2:** Tree characteristics of all larch sample trees in each plot, with arithmetic means (±standard deviation) of diameter at breast height (DBH), crown ratio (CR), branch height (BH), canopy depth (CD) and specific leaf area (SLA)

Plot number	DBH (cm)	CR (%)	BH (m)	CD (m)	SLA (cm^2^ g^−1^)
2	25.7 (±8.2)	65.5 (±8.5)	11.9 (±3.6)	11.4 (±3.7)	144.9 (±33.8)
3	22.2 (±6.3)	49.2 (±9.4)	14.1 (±2.6)	11.5 (±2.6)	120.8 (±36.6)
5	37.3 (±10.2)	32.1 (±5.1)	28.3 (±3.8)	14.7 (±3.8)	107.7 (±15.9)
6	32.8 (±6.0)	36.7 (±7.0)	25.6 (±3.5)	11.3 (±3.5)	135.7 (±23.0)
8	45.6 (±8.3)	49.3 (±9.3)	23.8 (±4.0)	14.7 (±4.0)	100.0 (±22.4)
9	45.3 (±9.3)	52.2 (±8.9)	28.0 (±5.5)	17.1 (±5.5)	106.6 (±19.0)
11	46.6 (±8.2)	39.9 (±5.0)	24.1 (±3.4)	13.1 (±3.4)	119.7 (±14.7)
12	36.9 (±9.1)	40.6 (±5.7)	24.2 (±4.1)	15.3 (±4.1)	106.2 (±14.2)
Overall	36.5 (±11.8)	45.7 (±12.4)	22.5 (±6.9)	13.6 (±4.3)	117.7 (±27.9)

### Variation of SLA

The SLA decreased within the crown from the bottom to the top, regardless of the recording year. No SLA differences were detected between pure and mixed stands as well as between the social positions (Table 3).

**Table 3:** Arithmetic means (±standard deviation) of specific leaf area for 120 individuals (*n*) of European larch in years 2012 and 2013, for mixed and pure stands, for the social position [dominated (1), intermediate (2), dominant (3)] and in three crown sections (lower, middle and upper crown section)

Year of sampling	*n*	Specific leaf area								
		Overall	Stand type		Social position			Crown section		
			Mixed	Pure	1	2	3	Lower	Middle	Upper
2012	60	118.1^a^ (±33.5)	122 (±36.4)	114 (±29.9)	124 (±39.7)	116 (±29.7)	114 (±29.8)	129^a^ (±36.9)	121^a^ (±30.5)	105^b^ (±28.5)
2013	60	117.3^a^ (±20.9)	114 (±16.4)	121 (±24.1)	120 (±23.1)	120 (±20.4)	112 (±18.3)	129^a^ (±20.3)	119^b^ (±18.4)	104^c^ (±15.8)
Mean	120	117.7 (±27.9)	118 (±28.5)	117 (±27.3)	122 (±32.4)	118 (±25.5)	113 (±24.6)	129^a^ (±29.7)	119^ab^ (±25.1)	105^b^ (±22.9)

All values are in cm^2^ g^−1^. Only the position within the crown varies significantly (*α* = 0.05). In this case, the means that are not significantly different (Scheffée-Test) are indicated by the same letter

The random effect of the trees within the plot was not significant and was not considered anymore in further data analysis.

Note that in Tables 2 and 3 the arithmetic means are reported. Kumer (2015, personal communication) found that 26 % of the leaf mass is located in the lower crown third, 42 % in the middle crown third and 32 % in the upper crown third. Considering the different proportion of leaf mass in the three crown thirds, the weighted mean specific leaf area is $\overline{SLA}_{\text{weighted}} = 117.1\, {\text{cm}}^{2}\, {\text{g}}^{ - 1}$.

An overall analysis with the natural logarithm of SLA as dependent variable, with the plot as random effect and all other variables and their interactions as fixed effects (Eq. 1) revealed no significant relationship at all. Non-significant variables were then stepwise eliminated from our analysis and parameters were re-estimated. Finally, only the variables, branch height and canopy depth were significant (*α* = 0.05).

The final equation is thus:

2 $$\ln \left( {\text{SLA}} \right)\left[ {{\text{cm}}^{2}\, {\text{g}}^{ - 1} } \right] = a + b\ln \left( {{\text{branch}} {\text{height}} \,\left[ m \right]} \right) + c\ln \left( {{\text{canopy}} {\text{depth}} \,\left[ m \right]} \right) + u$$

with *u*, the random effect of the plots (standard deviation = ±0.119), *a* the intercept and *b* and *c*, the estimated coefficients of the fixed effects, given in Table 4. About 22 % of the variation was explained by this equation.

**Table 4:** Summary of results from the final model (see Eq. 2), with the coefficients, their standard error (SE), degrees of freedom of the denominator (DF) and *p* value

Fixed effect	Coefficient	SE	DF	*p* value
Intercept	5.258888	0.3873	350	0.0000
ln(branch height)	−0.272955	0.0836	350	0.0012
ln(canopy depth)	0.123760	0.0565	350	0.0290

Besides these two variables, canopy depth and branch height, none of the others added significant information for SLA, indicating that the effects of age, altitude above sea level, DBH, tree height, etc. and their interactions are sufficiently described by the two variables left in the equation.

Please note that within a plot, canopy depth and branch height are strictly linearly related. Over all plots, however, they are nearly uncorrelated (*R*^2^ = 0.053) because of the different canopy heights (maximum tree height) of the plots (see Fig. 1; Table 1). Thus for the whole dataset, the effects of canopy depth and branch height are not confounded.

### Short shoot needles versus long shoot needles

As mentioned before we investigated differences between the SLA of the needles on short and long shoots from the samples of 2013. As a result, the needles on the short shoots had an approximately 4 cm^2^ g^−1^ higher SLA than those on the long shoots (*p* < 0.001). These differences were the most obvious in the lower section of the crown, but not significant in the uppermost crown section (Table 5).

**Table 5:** The mean differences between specific leaf area (ΔSLA) of the short and the long shoot needles: ΔSLA = SLA_short-shoot −_ SLA_long-shoot_ (cm^2^ g^−1^) and the standard deviations in different crown sections

	Crown section			
	Lower	Middle	Upper	All
Mean	8.46^a^	5.56^ab^	−0.644^b^	4.46
Standard deviation	±17.8	±17.7	±13.7	±16.9
*p* (>*t*)	0.00048	0.0181	0.7173	0.00049

The last line indicates the results of *t* tests for mean difference being 0

The means that are not significantly different (Scheffée-Test) are indicated by the same letter

## Discussion

European larch occupies a special position among European conifers with regard to its deciduous behaviour. The high value of SLA of our study of 117 cm^2^ g^−1^ with a standard deviation of ±27.9 cm^2^ g^−1^ is supported by the results of other studies, which have also found high values for SLA of larch. Gower and Richards (1990) report an SLA of European larch of 123 cm^2^ g^−1^, whereas Matyssek and Schulze (1987a, b) state an average mass per all-sided leaf area of 4.13 mg cm^−2^, resulting in a SLA (which is based on projected leaf area) of 121 cm^2^ g^−1^.

Wieser et al. (2013) studied the long-term impact of ozone on photosynthesis of tree species at the timber line. They conclude that the high sensitivity of European larch to O_3_ is a result of its high SLA of 125 cm^2^ g^−1^.

An older investigation by Burger (1945) with a sample of about 100 individuals of European larch, scattered all over Switzerland reports an average SLA of 152 cm^2^ g^−1^. With this exception, our result deviates only negligibly from previous reports, e.g. Gower and Richards (1990), Matyssek and Schulze (1987a, b) and Wieser et al. (2013).

### Variation of SLA

SLA varies within the crown, but not between stand type, data recording years and social position.

The variation within the crown can be confirmed with numerous studies, which have well documented the high influence of the needle/leaf position in other tree species.

Most of the authors interpret this result as an expression of the investigated species’ ability to cope with changing light. Hager and Sterba (1985) found this for Norway spruce; Abrams and Kubiske (1990) for different hardwood species; Chen and Klinka (1998) for *Larix orientalis*; Chen (1997) and Bond et al. (1999) for *Pseudotsuga menziesii* and *Pinus ponderosa*.

Poorter et al. (2006) found the response of larch to lower irradiance smallest compared to other tree species, and Marshall and Monserud (2003) did not find a strong shading effect on SLA. However, understanding canopy depth as a proxy for light availability within the crown, we could proof such an influence on SLA (*p* = 0.0290). A review, dealing with the within-canopy variations in leaf structural, chemical and physiological traits, reports on the results of 292 studies for 304 taxa and concludes that the light-dependent increases in foliage photosynthetic capacity per area are surprisingly similar in different plant functional types. They however differ fundamentally in the way of their control by constituent traits (Niinemets et al. 2015). Unfortunately this review does not comprise any study dealing with European larch and only four out of 292 studies dealing with other larch species.

**Fig. 2:**
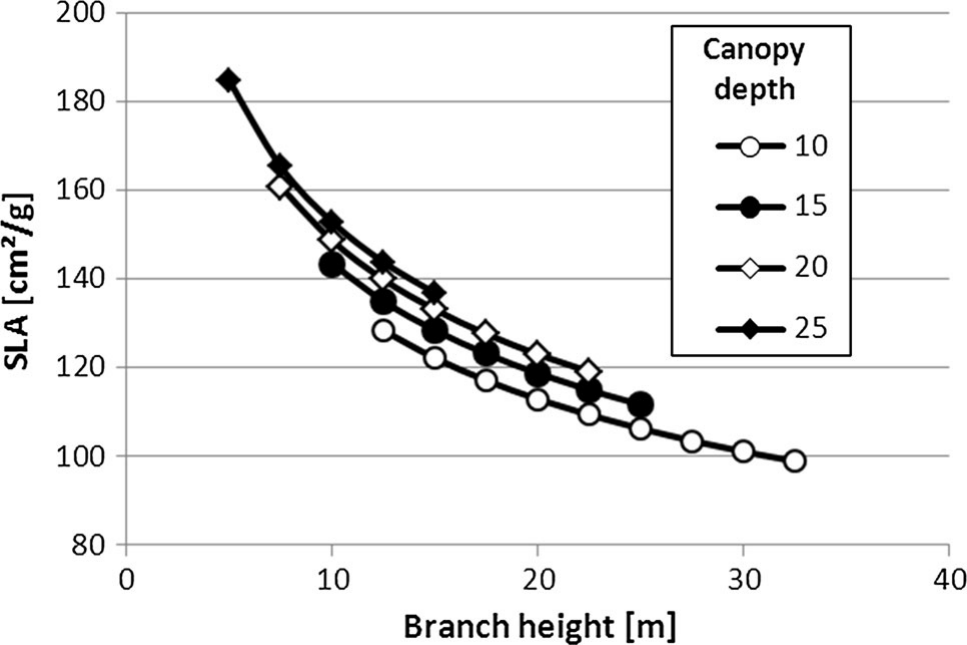
Specific leaf area depending on the branch height and canopy depth (10, 15, 20 and 25 m) for European larch

However, we also found that branch height had an additional significant influence on SLA (*p* = 0.0032). This agrees well with Marshall and Monserud’s (2003) interpretation of the influence of the gravitational component of the water potential, falling with increasing branch height (Fig. 2) for three different tree species in Idaho as a cause for the decrease of SLA from the bottom to the top of the crown. This interpretation may also explain the frequently found effect of drought on decreasing SLA (e.g. Phillips and Riha 1993; van Hees 1997; Ibrahim et al. 1998; Myers et al. 1998). Since European larch (together with Norway spruce) has been found most susceptible to drought (Lévesque et al. 2013), this additionally may explain why we found this relationship highly significant.

The *R*^2^, for the mixed-effects model was quite weak (*R*^2^ = 0.22). A reason could be the rather small variation of our SLA (coefficient of variation = ±24 %), due to the absence of shade needles. This compares well to the study of Marshall and Monserud (2003) where the *R*^2^ for the relationship between branch height and SLA is also weakest for ponderosa pine (*R*^2^ = 0.22), where the total variation of SLA is smallest (coefficient of variation = 18 %).

When comparing short shoot needles with long shoot needles, we found a slightly higher average SLA at short shoot needles than on long shoot needles (about 4 cm^2^ g^−1^; *p* ≪ 0.001). Although highly significant, the difference between the SLA of short shoot needles and the SLA of long shoot needles was only about 3 %. Neglecting this difference is even more justified because Burger (1945) claims that larch has much more short than long shoot needles, and therefore the needles on long shoots may be neglected.

## Conclusions

- Mean SLA is approximately 117 cm^2^ g^−1^. Compared to other conifer tree species like Norway spruce or Scots pine it is two to four times higher. This is supported by the studies of Gower and Richards (1990), Matyssek and Schulze (1987a, b) and Wieser et al. (2013).
- Branch height as well as canopy depth have a significant influence on the SLA of European larch. This indicates that the hydraulic limitations and lower water potentials in greater branch heights, as well as the decrease in light availability with increasing crown depth are influencing the SLA of European larch.
- The SLA of short and long shoot needles differs statistically significant, but this difference is only about 3 %.

### **Authors contribution statement**

All authors contributed equally.

## Abbreviations

SLA (cm^2^ g^−1^): Specific leaf area (leaf mass per projected leaf area)
DBH (cm): Diameter at breast height (1.3 m)
CS: Crown section (lower, middle, upper crown section)
ST: Stand type (mixed or pure)
SOC: Social position (dominated, intermediate, dominant)
